# Integrin β1-Mediated Cell–Cell Adhesion Augments Metformin-Induced Anoikis

**DOI:** 10.3390/ijms20051161

**Published:** 2019-03-07

**Authors:** Tingting An, Zhiming Zhang, Yuhuang Li, Jianqiao Yi, Wenhua Zhang, Deshi Chen, Juan Ao, Zhi-Xiong Xiao, Yong Yi

**Affiliations:** Center of Growth, Metabolism and Aging, Key Laboratory of Bio-Resource and Eco-Environment, Ministry of Education, College of Life Sciences, Sichuan University, Chengdu 610064, China; tingtinganscu@163.com (T.A.); zhangzmzzm@163.com (Z.Z.); lyh0824@foxmail.com (Y.L.); yijianqiao@126.com (J.Y.); 15940214408@163.com (W.Z.); chendeshi1991@hotmail.com (D.C.); Aoj338@nenu.edu.cn (J.A.); jimzx@scu.edu.cn (Z.-X.X.)

**Keywords:** metformin, ΔNp63α, integrin β1, cell adhesion, anoikis

## Abstract

Cell–cell adhesion plays an important role in regulation of cell proliferation, migration, survival, and drug sensitivity. Metformin, a first line drug for type 2 diabetes, has been shown to possess anti-cancer activities. However, whether cell–cell adhesion affects metformin anti-cancer activity is unknown. In this study, Microscopic and FACS analyses showed that metformin induced cancer cell–cell adhesion exemplified by cell aggregation and anoikis under glucose restriction. Furthermore, western blot and QPCR analyses revealed that metformin dramatically upregulated integrin β1 expression. Silencing of integrin β1 significantly disrupted cell aggregation and reduced anoikis induced by metformin. Moreover, we showed that p53 family member ΔNp63α transcriptionally suppressed integrin β1 expression and is responsible for metformin-mediated upregulation of integrin β1. In summary, this study reveals a novel mechanism for metformin anticancer activity and demonstrates that cell–cell adhesion mediated by integrin β1 plays a critical role in metformin-induced anoikis.

## 1. Introduction

Metformin has been widely used to treat type 2 diabetes for many years. In recent years, clinical and basic evidence indicates that metformin has biological activities beyond the treatment of diabetes, such as anti-aging [1,2] and anti-obesity effects [3,4]. Metformin was the first FDA-approved drug in a clinical anti-aging trial [5]. Moreover, accumulating evidence demonstrates that metformin has anti-cancer activities [6]. Metformin can inhibit cancer cell growth, survival, and metastasis [7,8]. In addition, latest studies demonstrate that metformin can improve tumor immune response [9] and modulation of cancer-related epigenetic modification [10]. Lots of researches demonstrate that activation of AMPK is the major mechanism of metformin anti-cancer [9,10,11]. Our previous study indicates that metformin-induced cancer cell anoikis is an AMPK-independent manner [12]. Currently, there are more than 300 FDA-approved clinical trials in assessment of metformin effects on cancer therapy (www.clinicaltrials.gov).

p63 is a member of p53 family proteins involved in multiple biological processes, including embryonic development, cell differentiation, proliferation, survival, and aging [13]. Due to alternative transcription starting from two sites and the alternative spicing at the C-termini, there are at least six major p63 protein isoforms [13]. ΔNp63α is the predominant isoform of p63 protein expresses in epithelial cells and is critical for epithelial development [13]. It is reported that ΔNp63α is frequently overexpressed in squamous cell carcinoma and promotes cell proliferation and tumor growth [14]. On the other hand, ΔNp63α is a common inhibitory target in oncogenic PI3K/Her2/Ras-induced cell migration and cancer metastasis [15]. Moreover, ΔNp63α is a master regulator of cell adhesion program, which regulates several adhesion molecules, including fibronectin 1, integrin α6, and integrin β4 [16]. Our previous study has demonstrated that metformin destabilizes ΔNp63α protein and inhibits cancer cell viability [12].

Cell–cell adhesion and cell-extracellular matrix (ECM) interaction are essential signals in regulation of cell proliferation, cell migration, survival and drug sensitivity [17,18]. When anchorage-dependent cells detach from the surrounding ECM, they often undergo anoikis, a form of programmed cell death [19]. Integrin β1 is a family member of integrins, playing an important role in maintaining cell-matrix adhesion [16,20]. Accumulating evidence indicates that integrin β1 is involved in tumor growth and cancer metastasis [21]. However, whether integrin β1 is involved in cell–cell adhesion or apoptosis/anoikis is unclear.

In this study, we showed that integrin β1-mediated cell–cell adhesion enhances metformin-mediated cell aggregation and anoikis under glucose restriction. ΔNp63α is a negative regulator of integrin β1, which plays a pivotal role in metformin-mediated upregulation of integrin β1. Taken together, this study demonstrates that ΔNp63α-integrin β1 axis is critical in metformin anticancer process and reveals a novel mechanism for metformin anticancer action.

## 2. Results

### 2.1. Metformin Induces Cancer Cell Aggregation/Detachment and Anoikis under Glucose Restriction

**Figure 1:**
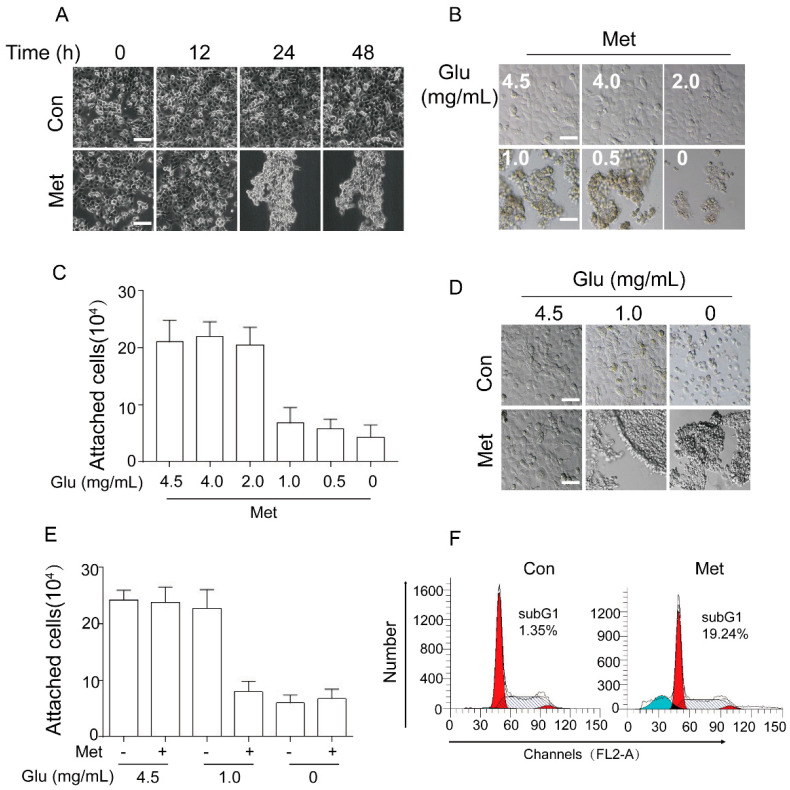
Metformin induces cell aggregation/detachment and anoikis under glucose restriction. (**A**) FaDu cells were treated with metformin (20 mM, and thereafter) for an indicated time (0, 12, 24, or 48 h) in DMEM containing 1.0 mg/mL glucose condition (DMEM-LG). Cell morphology was captured by phase contrast microscopy. (**B**,**C**) FaDu cells were treated with metformin for 24 h in DMEM containing an indicated concentration of glucose (4.5, 4.0, 2.0, 1.0, 0.5, or 0 mg/mL). Cell morphology was captured by phase contrast microscopy and representative pictures were shown (**B**). Attached cells were quantitated and presented (**C**). (**D**,**E**) FaDu cells were treated with or without metformin for 24 h in DMEM containing an indicated dose of glucose (4.5, 1.0, or 0 mg/mL) respectively. Representative images were shown (**D**). Attached cell were quantitated and presented (**E**). (**F**) FaDu cells were treated with or without metformin for 48 h in DMEM-LG. Both adherent and floating cells were subjected to FACS analyses. All experiments were performed three times in triplicates. Data were presented as means ± s.d. Scale bar = 50 µm.

We have previously shown that metformin inhibits expression of ΔNp63α resulting in human head and neck squamous cell carcinoma (HNSCC) FaDu cell anoikis under glucose restriction [12]. During the course of the study, we constantly noticed that metformin significantly induced cell aggregation prior to detachment under glucose restriction (DMEM containing 1.0 mg/mL glucose). As shown in Figure 1A, under glucose restriction, FaDu cells showed little cell detachment, whereas treatment with 20 mM metformin led to dramatic Cell Aggregation and Detachment (CAD), consistent with our previous report [12]. We then examined the impact of glucose concentration on metformin-induced CAD. As shown in Figure 1B,C, metformin-induced CAD only occurred at the condition of restricted glucose.

It has been reported that human hepatoma HepG2 cells can undergo detachment in the absence of glucose [22], we therefore examined the effects of no glucose on cell detachment in comparison to the effects of metformin. As shown in Figure 1D,E, cells grown in DMEM containing 1.0 mg/mL glucose exhibited no detachment, whereas cells grown in DMEM containing 0 mg/mL glucose exhibited cell detachment without cell aggregation. By contrast, cells grown in DMEM containing 0 mg/mL glucose in the presence of metformin exhibited dramatic CAD. These results indicate that metformin specifically induces CAD.

We next examined the effects of metformin on cell viability. As shown in Figure 1F, metformin significantly induced FaDu cell apoptosis under glucose restriction, consistent with our previous study [12]. Taken together, these results demonstrate that metformin can induce cancer cell detachment and apoptosis, a well-known phenomenon characterized as anoikis.

### 2.2. Metformin Induces Expression of Integrin β1 Resulting in Cell Aggregation and Promoting Anoikis

**Figure 2:**
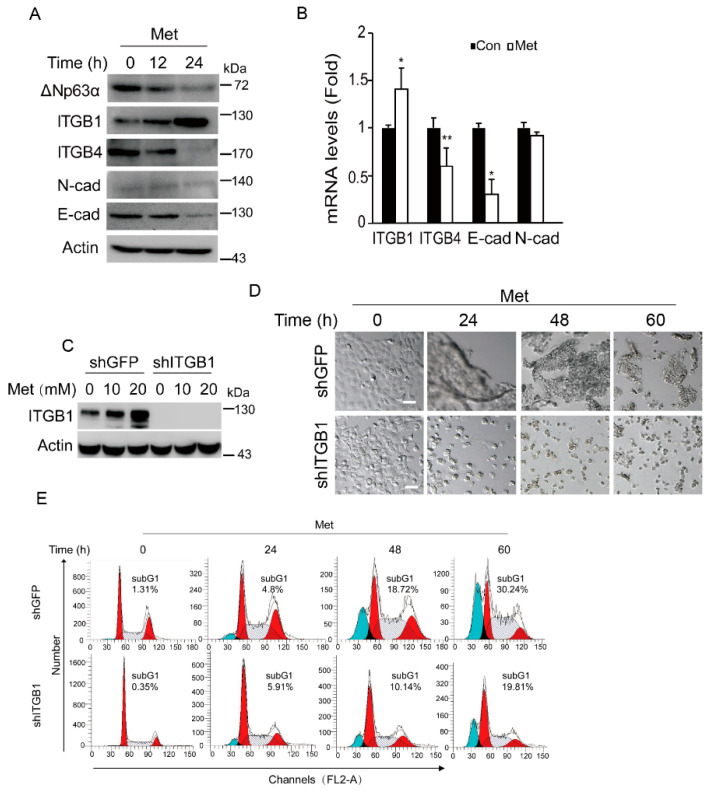
Metformin induces expression of integrin β1 resulting in cell aggregation and promoting anoikis. (**A**,**B**) FaDu cells were treated with metformin (20 mM, and thereafter) for an indicated time (0, 12, or 24 h) in DMEM-LG. Cells were subjected to western blot analyses (**A**) or subjected to Q-PCR analyses (cells were collected at 18 h) (**B**). Statistical analyses were based on Student’s *t*-test. Data were presented as means ± s.d. **. *p* < 0.05; *, *p* < 0.05. (**C**) FaDu cells stably expressing shITGB1 or shGFP were treated with metformin (0, 10, or 20 mM) in DMEM-LG for 24 h. Cells were subjected to western blot analyses. (**D**,**E**) FaDu cells stably expressing shITGB1 or shGFP were treated with metformin in DMEM-LG for an indicated time (0, 24, 48, or 60 h). Representative images were shown (**D**) and cells were subjected to FACS analyses (**E**, Red indicates G1 and G2 phases, blue marks the subG1 population (apoptotic cells), and the slash line denotes the S phase). Scale bars = 50 µm. For western blotting, all PVDF membranes derived from the transfers were cut at appropriate areas and reacted with an indicated primary antibody.

To dissect the molecular basis by which metformin induces CAD, we hypothesized that metformin may upregulate subset of cell adhesion molecules, such as integrin β1, integrin β4, N-cadherin, and E-cadherin. We first examined the impact of metformin on the protein levels of several cell-adhesion molecules. As shown in Figure 2A,B, metformin treatment led to a significant decrease in integrin β4 and E-cadherin at both protein and mRNA levels. Surprisingly, metformin dramatically upregulated expression of integrin β1 at both protein and mRNA levels (Figure 2A,B). Moreover, consistent with our previous data [12], metformin significantly inhibited ΔNp63α protein expression (Figure 2A).

Next, we examined whether integrin β1 plays a role in metformin-induced cell aggregation. As shown in Figure 2C,D, again, metformin significantly upregulated expression of integrin β1, concomitant with CAD. Silencing of integrin β1 completely eliminated metformin-induced cell aggregation. Importantly, ablation of integrin β1 clearly reduced metformin-induced apoptosis (Figure 2E). Taken together, these results indicate that integrin β1 plays an important role in metformin-induced cell aggregation and anoikis.

### 2.3. Metformin Upregulates Integrin β1 Expression via Inhibiting ΔNp63α

**Figure 3:**
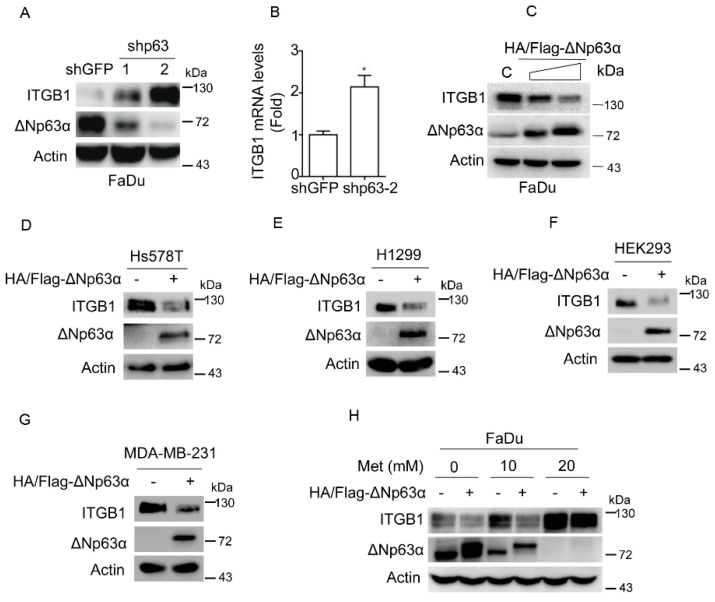
Metformin suppresses expression of ΔNp63α, resulting in upregulation of integrin β1. (**A**,**B**) FaDu cells stably expressing shp63-1, shp63-2 or shGFP were subjected to western blot analyses (**A**) or subjected to Q-PCR analyses (**B**). Data are presented as means ± s.d. *, *p* < 0.05. (**C**) FaDu cells stably overexpressing HA/Flag-ΔNp63α or vector were subjected to western blot analyses, with sample processing controls run on separate gels. (**D**–**G**) Ectopic expression of HA/Flag-ΔNp63α in Hs578T (**D**), H1299 (**E**), HEK293 (**F**), and MDA-MB-231 (**G**) cells were subjected to western blot analyses. (**H**) FaDu cells stably overexpressing HA/Flag-ΔNp63α or vector were treated with metformin (0, 10, or 20 mM) for 24 h in DMEM-LG. Cells were subjected to western blot analyses. All western blot membranes were cut at appropriate areas and reacted with an indicated primary antibody.

We then investigated the molecular mechanism by which metformin induces expression of integrin β1. We have previously shown that metformin can significantly inhibit ΔNp63α expression. Since p63 is a master regulator to transcriptionally regulate expression of cell adhesion molecules [16], we therefore examined the effects of ΔNp63α on integrin β1 expression. As shown in Figure 3A,B, silencing of ΔNp63α dramatically upregulated integrin β1 expression at both protein and mRNA levels. On the other hand, ectopic expression of ΔNp63α significantly downregulated integrin β1 protein expression in a dose-dependent manner (Figure 3C). Similar results are observed in other cell lines, including human breast cancer Hs578T and MDA-MB-231, human non-small cell lung cancer (NSCLC) H1299 and human embryonic kidney HEK293, all of which express high levels of endogenous integrin β1 (Figure 3D–G). Importantly, restoration of ΔNp63α expression effectively reversed metformin-induced upregulation of integrin β1 expression (Figure 3H). Together, these results demonstrate that ΔNp63α is a critical negative regulator of integrin β1 and is most likely responsible for metformin-mediated upregulation of integrin β1.

## 3. Discussion

Until now, it has been well established that metformin possesses anti-cancer activities. Currently, there are FDA-approved more than 300 clinical trials to assess effects of metformin on cancer therapy (www.clinicaltrials.gov.13/01/2019). In this study, we showed that metformin induces cancer cell anoikis. Furthermore, we found that integrin β1 is pivotal in both metformin-induced cell aggregation and anoikis. Moreover, we demonstrated that ΔNp63α is a negative regulator of integrin β1 and is responsible for metformin-mediated upregulation of integrin β1.

How does metformin upregulate integrin β1 expression? Our results clearly indicate that ΔNp63α is a pivotal negative regulator of integrin β1 expression and is responsible to metformin-mediated upregulation of integrin β1. However, in contrast to a possible positive role for p63 in regulation of integrin β1 as shown in MCF 10A cells [16], our data reproducibly show that expression of ΔNp63α leads to downregulation of integrin β1 in FaDu, Hs578T, H1299, HEK 293, and MDA-MB-231 cells. The reason for the discrepancy is not yet clear although it might be possible that there are some unknown factors affecting expression of integrin β1 in MCF 10A cells.

**Figure 4:**
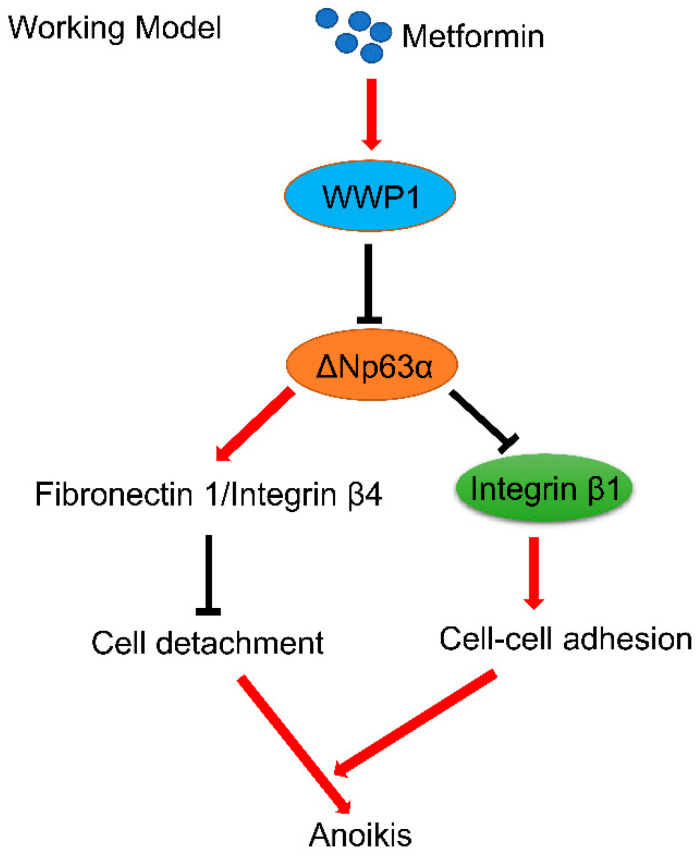
A working model of metformin-induced cancer cell anoikis. Metformin upregulates ubiquitin E3 ligase WWP1, leading to destabilization of ΔNp63α and downregulation of proteins involved in cell-matrix adhesion including integrin β4 and fibronectin 1, which in turn results in cell detachment [12]. On the other hand, metformin inhibits ΔNp63α expression resulting in upregulation of integrin β1, which in turn promotes cell–cell adhesion and cell aggregation. Both disruption of cell-matrix adhesion and enhanced cell–cell adhesion contribute to metformin-induced anoikis.

Cell–cell adhesion is fundamentally important for cell–cell communication involved in multiple facets of biological processes, including cell proliferation, migration, and survival [23]. Our previous study demonstrates that metformin destabilizes ΔNp63α via upregulating ubiquitin E3 ligase WWP1, leading to downregulation of proteins involved in cell-matrix adhesion including integrin β4 and fibronectin 1, which in turn results in cell detachment [12]. In this study, we further showed that metformin not only induces cell detachment but also induces cell aggregation, both of which significantly contribute to metformin-mediated anoikis (Figure 4).

E-cadherin is an adherent junction marker, critically important in maintaining epithelial cell–cell adhesion [24]. However, we found that metformin significantly inhibits E-cadherin expression at both mRNA and protein levels, indicating that E-cadherin is unlikely to be responsible for metformin-mediated cell aggregation. On the other hand, integrin β1 is a transmembrane receptor which has been shown as a critical protein in maintaining cell-extracellular matrix (ECM) adhesion [16,20]. Most interestingly, we found that metformin can transcriptionally upregulate integrin β1 expression, which in turn plays a causative role in both metformin-mediated cell aggregation and anoikis. Together, our findings demonstrate that integrin β1 is not only important in maintaining cell-matrix adhesion but also is critical in cell–cell adhesion. Furthermore, our data clearly indicates that integrin β1-mediated cell–cell adhesion plays an important role in metformin-induced anoikis. It would be interesting to further investigate whether cell–cell adhesion plays a role in cell viability under pathophysiological conditions in vivo.

Most notably, it is reported that circulating tumor cell clusters exhibit enhanced metastatic potential due to increased cancer cell stemness and/or increased anoikis-resistance [25,26]. Therefore, it is conceivable that circulating tumor cell clusters might be more sensitive to metformin. Conceptually, integrin β1 could be a potential target for cancer therapy.

## 4. Materials and Methods

### 4.1. Cell Culture and Drug Treatment

Human head and neck squamous cell carcinoma (HNSCC) FaDu cells, HEK293 cells, HEK293T cells, human breast cancer MDA-MB-231 and Hs578T cells, and human non-small cell lung cancer (NSCLC) H1299 cells were purchased from ATCC and maintained in DMEM medium (GIBCO, Rockville, MD, USA) containing 10% fetal bovine serum (FBS; Hyclone, Logan, UT, USA), 100 units/mL penicillin (GIBCO) and 100 µg/mL streptomycin (GIBCO). Cells were grown in a humidified 37 °C incubator under a 5% CO_2_ atmosphere. Cells at 75–85% confluence were treated with an indicated chemical compound. Metformin (PHR1084) was purchased from Sigma-Aldrich (St Louis, MO).

### 4.2. Plasmids Transfection, Lentiviral Infection, and RNA Interference

Cells at 80% confluence were transfected using Lipofectamine 2000 transfection reagent (Invitrogen, Carlsbad, CA, USA). Recombinant lentiviruses were generated by transfecting HEK293T cells with psPAX2 and pMD2.G packaging plasmids and lentiviral expression plasmid human HA/Flag-ΔNp63α using Lipofectamine 2000. Virus were collected at 60 h post-transfection. Cells at 30% confluence supplemented with 10 µg/mL polybrene were infected with recombinant lentivirus encoding an empty vector or expression plasmid, followed by 12 h incubation at 37 °C with 5% CO_2_. Lentiviral-based shRNAs specific for green fluorescent protein (GFP, GAAGCAGCACGACTTCTTC), integrin β1(GGAATGCCTACTTCTGCAC) and human p63 (GAGTGGAATGACTTCAACTTT; CCGTTTCGTCAGAACACACAT) were constructed as described [27].

### 4.3. Western Blot Analyses

Cells were lysed in EBC250 lysis buffer (250 mM NaCl, 50 mM Tris pH 8.0, 0.5% Nonidet *p*-40, 50 mM NaF, 2 µg/mL aprotinin, 1 mM phenylmethylsulfonyl fluoride and 2 µg/mL leupeptin). Whole cell lysates (50 µg) were separated by SDS-PAGE, and then transferred to PVDF membranes (Millipore, Darmstadt, Germany). Membranes were blocked in 5% milk in TBS-T buffer and then hybridized to an appropriate primary antibody and HRP-conjugated goat anti-mouse IgG (1:3000, Santa Cruz Biotech (Santa Cruz, CA, USA), sc-2005) or goat anti-rabbit IgG (1:3000, Santa Cruz Biotech, sc-2004) for subsequent detection by enhanced chemiluminescence kit (WBKLS0500, Millipore). Specific antibodies for p63 (sc-8431,1:200) or actin (sc-1615, 1:1000) were purchased from Santa Cruz Biotech. Antibodies for N-cadherin (14215, 1:1000) or Integrin β4 (4704, 1:1000) were purchased from Cell Signaling Technology (Danvers, MA, USA). Antibodies for Integrin β1 (ab52971, 1:1000) or E-cadherin (ab40772, 1:10000) were purchased from Abcam (Cambridge, MA, USA).

### 4.4. Quantitative PCR

Total RNA was extracted from cells using RNeasy plus Mini Kit (cat# 74134, QIAGEN) and reverse-transcribed. Q-PCR was performed for Integrin β1 (F: GCCTTACATTAGCACAACACC; R: CATCTCCAGCAAAGTGAAAC), Integrin β4 (F: CACCGCGTGCTAAGCACAT; R: TGTGGTCGAGTGAGTGTTCTG), E-cadherin (F: GGATGTGCTGGATGTGAATG; R: CACATCAGACAGGATCAGCAGAA), N-cadherin (F: CTCCTATGAGTGGAACAGGAACG; R: TTGGATCAATGTCATAATCAAGTGCTGTA) and GAPDH (F: GGGGAGCCAAAAAGGGTCATCATCT; R: GAGGGGCCATCCACAGTCTTCT). The Q-PCR reactions were carried out in CFX-960 Real time PCR System (Bio-Rad, Saint-Laurent, QC, Canada) and using Bio-Rad SoFast Eva-Green Supermix (Bio-Rad) according to the manufacturer’s instructions. Relative quantitation values were calculated using the ΔΔCt method.

### 4.5. FACS Assay

Cells were grown in 6-well plates to approximately 80% confluence prior to treatment with metformin. Both floating and adherent cells were collected and trypsinized to obtain individual cell, which were then fixed in 75% ethanol at 4 °C overnight. Cells were stained with 50 µg/mL propidium iodide (PI)/80 µg/mL RNase A at 37 °C for 1 h. Cells were then subjected to FACS analysis by FACScalibur flow cytometer (Becton Dickson, Bedford, MA, USA).

### 4.6. Statistical Analysis

Quantitative data were analyzed statistically using Student’s *t*-test to assess significance. All experiments were performed three times in triplicates. Data are presented as means ± s.d.

## Abbreviations

ECM	Cell-extracellular matrix
CAD	Cell Aggregation and Detachment
HNSCC	Human head and neck squamous cell carcinoma
NSCLC	Human non-small cell lung cancer
