# Correction: An et al. Integrin β1-Mediated Cell−Cell Adhesion Augments Metformin-Induced Anoikis. *Int. J. Mol. Sci.* 2019, *20*, 1161

**DOI:** 10.3390/ijms262010014

**Published:** 2025-10-15

**Authors:** Tingting An, Zhiming Zhang, Yuhuang Li, Jianqiao Yi, Wenhua Zhang, Deshi Chen, Juan Ao, Zhi-Xiong Xiao, Yong Yi

**Affiliations:** Center of Growth, Metabolism and Aging, Key Laboratory of Bio-Resource and Eco-Environment, Ministry of Education, College of Life Sciences, Sichuan University, Chengdu 610064, China; tingtinganscu@163.com (T.A.); zhangzmzzm@163.com (Z.Z.); lyh0824@foxmail.com (Y.L.); yijianqiao@126.com (J.Y.); 15940214408@163.com (W.Z.); chendeshi1991@hotmail.com (D.C.); Aoj338@nenu.edu.cn (J.A.); jimzx@scu.edu.cn (Z.-X.X.)

In the original publication [1], there were two mistakes in Figure 2 and Figure 3 as published. 1. In Figure 2D, the image shown in panel shGFP−24 h is incorrect; 2. In Figure 3C, the western blot images are incorrect. The corrected Figure 2 and Figure 3 appear below. The authors state that the scientific conclusions are unaffected. This correction was approved by the Academic Editor. The original publication has also been updated.

## Figures and Tables

**Figure 2:**
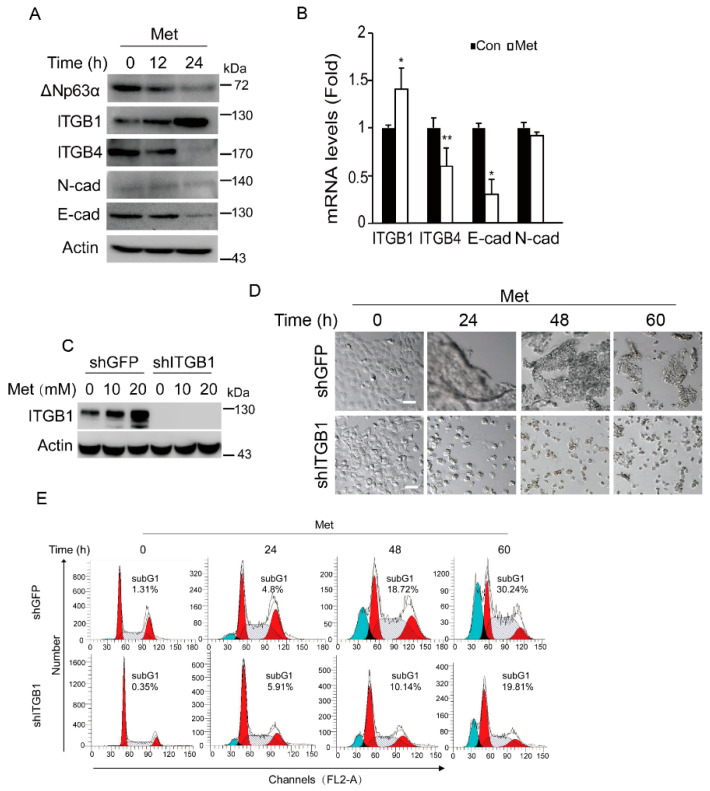
Metformin induces expression of integrin β1 resulting in cell aggregation and promoting anoikis. (**A**,**B**) FaDu cells were treated with metformin (20 mM, and thereafter) for an indicated time (0, 12, or 24 h) in DMEM-LG. Cells were subjected to western blot analyses (**A**) or subjected to Q-PCR analyses (cells were collected at 18 h) (**B**). Statistical analyses were based on Student’s *t*-test. Data were presented as means ± s.d. **. *p* < 0.05; *, *p* < 0.05. (**C**) FaDu cells stably expressing shITGB1 or shGFP were treated with metformin (0, 10, or 20 mM) in DMEM-LG for 24 h. Cells were subjected to western blot analyses. (**D**,**E**) FaDu cells stably expressing shITGB1 or shGFP were treated with metformin in DMEM-LG for an indicated time (0, 24, 48, or 60 h). Representative images were shown (**D**) and cells were subjected to FACS analyses (**E**, Red indicates G1 and G2 phases, blue marks the subG1 population (apoptotic cells), and the slash line denotes the S phase). Scale bars = 50 µm. For western blotting, all PVDF membranes derived from the transfers were cut at appropriate areas and reacted with an indicated primary antibody.

**Figure 3:**
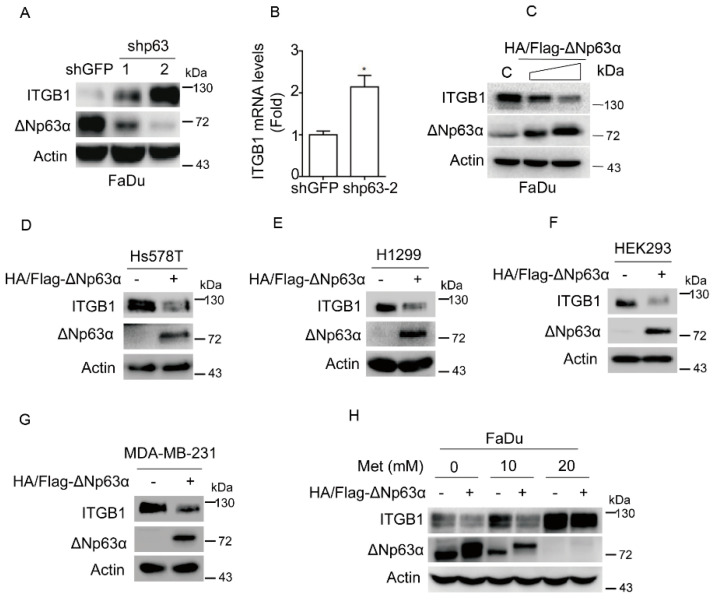
Metformin suppresses expression of ΔNp63α, resulting in upregulation of integrin β1. (**A**,**B**) FaDu cells stably expressing shp63-1, shp63-2 or shGFP were subjected to western blot analyses (**A**) or subjected to Q-PCR analyses (**B**). Data are presented as means ± s.d. *, *p* < 0.05. (**C**) FaDu cells stably overexpressing HA/Flag-ΔNp63α or vector were subjected to western blot analyses, with sample processing controls run on separate gels. (**D**–**G**) Ectopic expression of HA/Flag-ΔNp63α in Hs578T (**D**), H1299 (**E**), HEK293 (**F**), and MDA-MB-231 (**G**) cells were subjected to western blot analyses. (**H**) FaDu cells stably overexpressing HA/Flag-ΔNp63α or vector were treated with metformin (0, 10, or 20 mM) for 24 h in DMEM-LG. Cells were subjected to western blot analyses. All western blot membranes were cut at appropriate areas and reacted with an indicated primary antibody.
